# Machine learning-based risk prediction model for postoperative abdominal distension after spinal surgery under general anesthesia

**DOI:** 10.3389/fmed.2026.1871136

**Published:** 2026-09-04

**Authors:** Chanli Wu, Liju Xie, Shihao Zhou, Yingjie Hu, Xialin Li, Zheng Huang, Dazhi Yang, Xihong Mou

**Affiliations:** 1 Department of Orthopaedics, Shenzhen Nanshan People's Hospital, Shenzhen, China; 2 Graduate School, Qinghai University, Xining, China; 3 Department of Orthopaedics, Affiliated Nanshan Hospital of Shenzhen University, Shenzhen, China

**Keywords:** abdominal distension, general anesthesia, machine learning, risk prediction, spinal surgery

## Abstract

**Background:**

Early identification of patients at risk of postoperative abdominal distension after spinal surgery remains clinically difficult. Machine learning may improve risk prediction by integrating routinely available perioperative information.

**Objective:**

This study aimed to develop and internally validate an interpretable machine learning-based predictive model for postoperative abdominal distension after spinal surgery under general anesthesia and to identify clinically relevant predictors for early nursing risk stratification.

**Methods:**

This retrospective single-center prediction model study included 1,234 patients who underwent spinal surgery under general anesthesia. Seventeen routinely recorded perioperative and early postoperative variables were evaluated, including postoperative ADL category(ADL), PCA, and postoperative antibiotic use (yes/no). The data were split using stratified sampling into training (80%) and internal test (20%) sets. Missing-value imputation, scaling, five-fold cross-validated grid search, and model fitting were performed within the training workflow. Logistic regression(LR), RF, support vector machine(SVM), and gradient boosting (GB)models were evaluated using discrimination, calibration, Brier score, decision curve analysis, and permutation importance. Exact TreeSHAP analysis was used to interpret global and patient-specific contributions in the highest-AUC model.

**Results:**

Postoperative abdominal distension occurred in 138 of 1,234 patients (11.18%). In the internal test set (*n* = 247; 28 events), random forest (RF) had the highest AUC (0.775, 95% CI 0.650–0.880), followed by GB (0.746, 95% CI 0.627–0.854), SVM (0.737, 95% CI 0.635–0.831), and LR (0.723, 95% CI 0.616–0.821). The AUC difference between RF and GB was statistically significant (*P* = 0.039); all other pairwise comparisons were not significant. TreeSHAP ranked PCA (mean absolute SHAP value, 0.041), surgical drainage tube placement (0.024), and operative time (OT)(0.023) as the leading global contributors. PCA use and drainage tube placement generally shifted predictions toward higher PAD risk, whereas higher postoperative ADL values generally shifted predictions toward lower risk.

**Conclusion:**

The models showed moderate internal discrimination but materially different sensitivity–specificity trade-offs. Permutation importance and TreeSHAP consistently identified PCA and drainage tube placement as leading random-forest predictors, while TreeSHAP additionally showed the direction and patient-specific magnitude of each contribution. These model explanations are predictive rather than causal. Prospective temporal validation, external validation, and threshold selection are required before bedside use.

## 1 Introduction

As the global burden of spinal disorders continues to increase, the number of spinal surgical procedures has grown substantially (1, 2). Postoperative abdominal complications, particularly abdominal distension, remain common and clinically challenging among patients undergoing spinal surgery under general anesthesia (3). General anesthesia can transiently impair gastrointestinal motility, leading to acute gastrointestinal dysfunction manifested as nausea, vomiting, constipation, delayed flatus, and intolerance of oral intake (4–6). Abdominal distension and nausea are among the most frequently reported symptoms in this clinical setting, with reported incidence varying substantially according to procedure and outcome definition. These complications may increase patient discomfort, delay postoperative recovery, prolong hospitalization, and increase healthcare resource use (7, 8).

In this study, postoperative abdominal distension (PAD) was defined as new-onset abdominal fullness or visible abdominal distension documented after surgery, with or without delayed passage of flatus or stool, nausea, vomiting, or intolerance of oral intake. PAD overlaps with postoperative gastrointestinal dysfunction and may represent an early or mild manifestation within the spectrum of postoperative ileus. Clinically, PAD may increase patient discomfort, delay oral intake and ambulation, prolong hospitalization, and increase nursing workload and healthcare resource use (3, 6, 8, 9).

Numerous studies have examined risk factors for PAD, including sex, age, surgical level, operative duration(OD), body mass index (BMI), blood loss(BL), opioid exposure,comorbidities and electrolyte disturbances (ED) (9–14). However, many existing risk assessment approaches rely on conventional statistical models with a limited number of variables. Such models may not fully capture nonlinear relationships or interactions among perioperative factors (12, 15–19). This study aimed to develop and internally validate machine-learning models using routinely recorded perioperative and early postoperative information, to identify risk factors for postoperative abdominal distension in patients receiving general anesthesia for spinal surgery.

## 2 Data and methods

This retrospective single-center prediction model study was reported in accordance with the TRIPOD+AI guidance for clinical prediction models using regression, artificial intelligence, or machine learning methods (20, 21).

### 2.1 Data preprocessing and missing data handling

The maximum variable-specific missingness was 3.0% (37/1,234 for postoperative ADL), and missingness for all other predictors was lower. Missing data were assumed to be missing at random and handled using multiple imputation by chained equations (MICE), with five completed datasets generated (22, 23). To prevent information leakage, imputation models were fitted using the training data within each analysis workflow and then applied to the corresponding internal test data; outcome values in the internal test set were not used during imputation. The complete preprocessing and modeling workflow was repeated in each imputed dataset, and patient-level predicted probabilities were averaged across imputations before test-set performance was calculated. Continuous predictors were standardized using parameters estimated from the training data, whereas binary and ordinal predictors retained their prespecified coding. Sex, age, BMI (≥24 kg/m²), postoperative ADL (≤40, 41–60, or >60), PCA, and postoperative antibiotic use were coded from the electronic health records.

### 2.2 Ethical approval

This study was conducted in accordance with the World Medical Association Declaration of Helsinki, as amended by the 75th WMA General Assembly in Helsinki, Finland, in October 2024 (17). Ethical approval was obtained from the Medical Ethics Committee of Shenzhen Nanshan People's Hospital (No. KY2022K080; approval date: 20240731), and written informed consent was obtained from all participants.

### 2.3 Study population and data collection

Clinical data were retrospectively collected from 1,234 patients who underwent spinal surgery under general anesthesia at Shenzhen Nanshan People's Hospital from 1 January 2022 to 31 December 2024. Seventeen candidate predictors were analyzed: sex, age, BMI ≥24 kg/m², smoking, drinking, OT, BL, urinary catheterization(UC), hypertension, diabetes mellitus(DM), cardiovascular disease(CD), serum potassium(K^+^), serum sodium(Na^+^), drainage tube placement, ADL, postoperative antibiotic use (yes/no), and postoperative PCA use(PCA). Postoperative antibiotic duration was not included. Because postoperative ADL, PCA, and antibiotic use occur after surgery, the resulting models were interpreted as early postoperative surveillance models rather than purely preoperative prediction models.

### 2.4 Inclusion and exclusion criteria

The inclusion criteria were as follows: (I) adult patients who underwent spinal surgery under general anesthesia during the study period; (II) availability of perioperative nursing and anesthesia records; (III) ability to report postoperative gastrointestinal symptoms; and (IV) provision of written informed consent or documented consent according to institutional requirements. These criteria were chosen to ensure comparable anesthesia exposure, temporal availability of predictors, and reliable symptom assessment (3, 4, 6, 11). The exclusion criteria were as follows: (I) pre-existing intestinal diseases, including chronic intestinal obstruction, inflammatory bowel disease, or severe constipation requiring regular treatment; (II) severe mental disorders or cognitive impairment that prevented reliable symptom reporting; (III) incomplete key outcome documentation; and (IV) reoperation or emergency surgery with substantially different perioperative pathways. These exclusions were applied to reduce outcome misclassification and confounding from baseline gastrointestinal dysfunction (9–11).

### 2.5 Sample collection and laboratory measurements

After an overnight fast, 5 mL of venous blood was collected from the antecubital vein of each participant into anticoagulant vacuum tubes for laboratory analysis. The samples were centrifuged at 3,000 rpm for 10 min, after which serum was separated and stored at 2–10°C until analysis. Na^+^ and potassium levels were measured using a VITROS 5,600 Integrated System automatic biochemical analyzer (Ortho Clinical Diagnostics/Johnson & Johnson, Raritan, NJ, USA). The reference ranges were 135–145 mmol/L for Na^+^ and 3.5–5.5 mmol/L for K^+^.

### 2.6 Model development and evaluation

All analyses were conducted in Python. Candidate predictors were prespecified according to the supplied data, prior literature, clinical plausibility, nursing relevance, and the requested perioperative/early postoperative scope (11, 13, 14, 24). The dataset was partitioned into a training set (80%; *n* = 987, 110 events) and an internal test set (20%; *n* = 247, 28 events) using stratified sampling with random seed 42. All 17 prespecified predictors were retained. Imputation, continuous-variable standardization, hyperparameter tuning, and model fitting were performed without using the internal test outcomes.

Four classifiers—LR, RF, SVM, and GB—were trained (25–27). Hyperparameters were selected by grid search using five-fold stratified cross-validation in the training set and mean cross-validated AUC as the optimization criterion. The evaluated grids and selected values are reported in Supplementary Table S1. Test-set performance was quantified using AUC, accuracy, precision, recall, F1 score, calibration plots, Brier score, and decision curve analysis (28, 29). Classification metrics used each estimator's native default decision rule. Test-set 95% confidence intervals were estimated with 2,000 stratified bootstrap resamples. AUCs were compared using the paired DeLong test (30). Random-forest permutation importance was calculated as the mean decrease in test-set AUC across 100 repeated feature permutations.

For interpretation of the highest-AUC random-forest model, exact tree-path-dependent SHAP (SHapley Additive exPlanations; TreeSHAP) values were computed from the fitted scikit-learn tree structures for the positive-class probability in every internal-test observation (31). Positive TreeSHAP values increased the predicted probability of postoperative abdominal distension, whereas negative values decreased it. Global importance was summarized by the mean absolute TreeSHAP value, and a beeswarm plot displayed the distribution and direction of contributions across the test cohort. Patient-specific waterfall plots decomposed the prediction for observations nearest the 90th and 10th percentiles of predicted risk. We verified exact additivity by reconstructing every test-set probability as the random-forest expected value plus the sum of its feature-level TreeSHAP values.

### 2.7 Statistical analysis

Descriptive statistics were used to summarize the two outcome groups. Continuous variables were reported as mean ± standard deviation or median [interquartile range], and categorical variables as frequency and percentage. Between-group comparisons used the Mann–Whitney *U*-test for continuous variables and the chi-square test or Fisher exact test for categorical variables, as appropriate. All model comparisons were conducted on the same internal test observations. Two-sided *P* < 0.05 was considered statistically significant. The complete analytical workflow, hyperparameter grids, selected hyperparameters, and software versions are reported to support reproducibility (Supplementary Table S2).

## 3 Results

### 3.1 Baseline characteristics of the dataset

Postoperative abdominal distension occurred in 138 of 1,234 patients (11.18%). In unadjusted comparisons, BL, CD, drainage tube placement, postoperative antibiotic use, PCA, and ADL differed between outcome groups (Table 1). The training set contained 987 patients (110 events), and the internal test set contained 247 patients (28 events).

**Table 1:** Characteristics of patients with and without postoperative abdominal distension.

Variable	Non-distension (*n* = 1,096)	Distension (*n* = 138)	Statistic	*P*-value
OT(min)	154.8 [105.9, 199.8]	150.0 [111.2, 195.0]	*U* = 73,934	0.842
BL, mL	50.0 [20.0, 200.0]	100.0 [50.0, 203.8]	*U* = 59,624	<0.001
K^+^, mmol/L	3.96 ± 0.33	3.90 ± 0.33	*U* = 80,354	0.060
Na^+^, mmol/L	140.50 ± 2.32	140.15 ± 2.78	*U* = 79,246	0.144
Male sex	619 (56.5%)	79 (57.2%)	*χ*^2^ = 0.006	0.936
Age≥60 years	377 (34.4%)	44 (31.9%)	χ^2^ = 0.242	0.623
BMI ≥24 kg/m^2^	584 (53.3%)	78 (56.5%)	χ^2^ = 0.395	0.530
Smoking	215 (19.8%)	25 (18.1%)	χ^2^ = 0.119	0.730
Drinking	60 (5.5%)	8 (5.8%)	χ^2^ = 0.000	1.000
UC	875 (79.8%)	119 (86.2%)	χ^2^ = 2.805	0.094
Hypertension	263 (24.0%)	33 (23.9%)	χ^2^ = 0.000	1.000
DM	157 (14.3%)	17 (12.3%)	χ^2^ = 0.258	0.611
CD	0 (0.0%)	4 (2.9%)	Fisher exact	<0.001
Surgical drainage tube	474 (43.2%)	96 (69.6%)	χ^2^ = 33.103	<0.001
Postoperative antibiotic use	1,007 (91.9%)	134 (97.1%)	χ^2^ = 4.076	0.043
PCA	497 (45.3%)	107 (78.1%)	χ^2^ = 50.982	<0.001
Postoperative ADL ≤40	517 (48.8%)	77 (55.8%)	χ^2^ = 13.976	<0.001
Postoperative ADL 41–60	445 (42.0%)	61 (44.2%)		
Postoperative ADL >60	97 (9.2%)	0 (0.0%)		

Continuous data are mean ± SD or median [IQR]; categorical data are *n* (%). ADL = activities of daily living; BMI = body mass index; PCA = patient-controlled analgesia. Percentages use available observations; postoperative ADL was categorized as ≤40, 41–60, or >60.

### 3.2 Predictive performance of different machine-learning models

**Figure 1:**
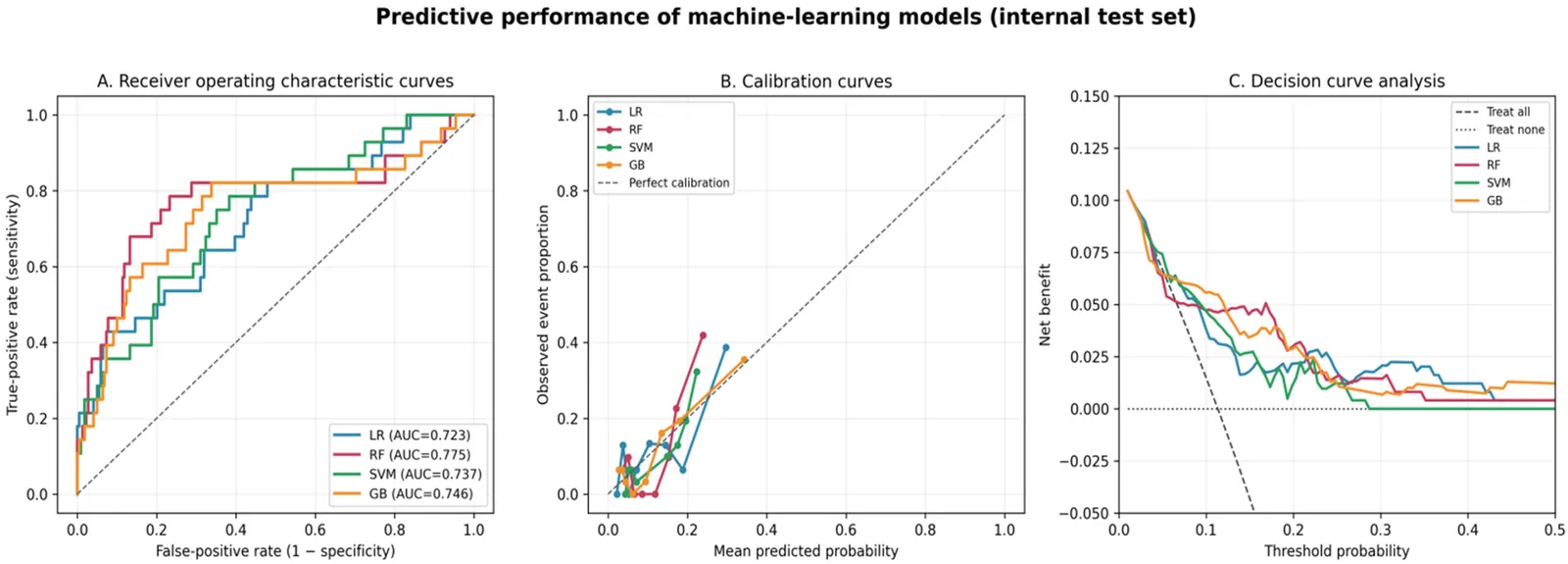
Internal test-set evaluation of LR, RF, SVM, and GB: **(A)** receiver operating characteristic curves, **(B)** calibration curves, and **(C)** decision curve analysis.

RF achieved the highest test-set AUC (0.775, 95% CI 0.650–0.880), followed by GB (0.746, 95% CI 0.627–0.854), SVM (0.737, 95% CI 0.635–0.831), and LR (0.723, 95% CI 0.616–0.821) (Table 2 and Figure 1). Calibration plots showed some deviation from the ideal line, and decision-curve patterns varied across clinically relevant thresholds. RF had a significantly higher AUC than GB (difference 0.029, 95% CI 0.001–0.057; *P* = 0.039); the other five pairwise comparisons were not statistically significant (Table 3).

**Table 2:** Internal test-set performance of the four machine-learning models.

Model	Accuracy (95% CI)	AUC (95% CI)	Precision	Recall	F1 score (95% CI)	Brier score
LR	0.891 (0.887–0.899)	0.723 (0.616–0.821)	1.000	0.036	0.069 (0.000–0.194)	0.088
RF	0.891 (0.887–0.899)	0.775 (0.650–0.880)	1.000	0.036	0.069 (0.000–0.194)	0.088
SVM	0.571 (0.510–0.632)	0.737 (0.635–0.831)	0.185	0.821	0.303 (0.248–0.356)	0.093
GB	0.899 (0.883–0.915)	0.746 (0.627–0.854)	0.800	0.143	0.242 (0.065–0.432)	0.086

Confidence intervals were obtained from 2,000 stratified bootstrap resamples. Classification metrics use each estimator's native default decision rule; therefore, SVM classification is based on its decision-function boundary rather than a 0.50 calibrated-probability cutoff.

Pairwise AUC comparisons are reported in Table 3.

**Table 3:** Pairwise deLong comparisons of internal test-set AUCs.

Model comparison	AUC difference	SE	*Z*	95% CI	*P*-value
LR vs. RF	−0.052	0.035	−1.489	−0.120 to 0.016	0.137
LR vs. SVM	−0.014	0.013	−1.056	−0.039 to 0.012	0.291
LR vs. GB	−0.023	0.032	−0.707	−0.086 to 0.040	0.480
RF vs. SVM	0.038	0.030	1.284	−0.020 to 0.096	0.199
RF vs. GB	0.029	0.014	2.059	0.001 to 0.057	0.039
SVM vs. GB	−0.009	0.028	−0.324	−0.063 to 0.045	0.746

A positive difference indicates that the first model had the larger AUC. LR = logistic regression; RF = random forest; SVM = support vector machine; GB = gradient boosting.

**Figure 2:**
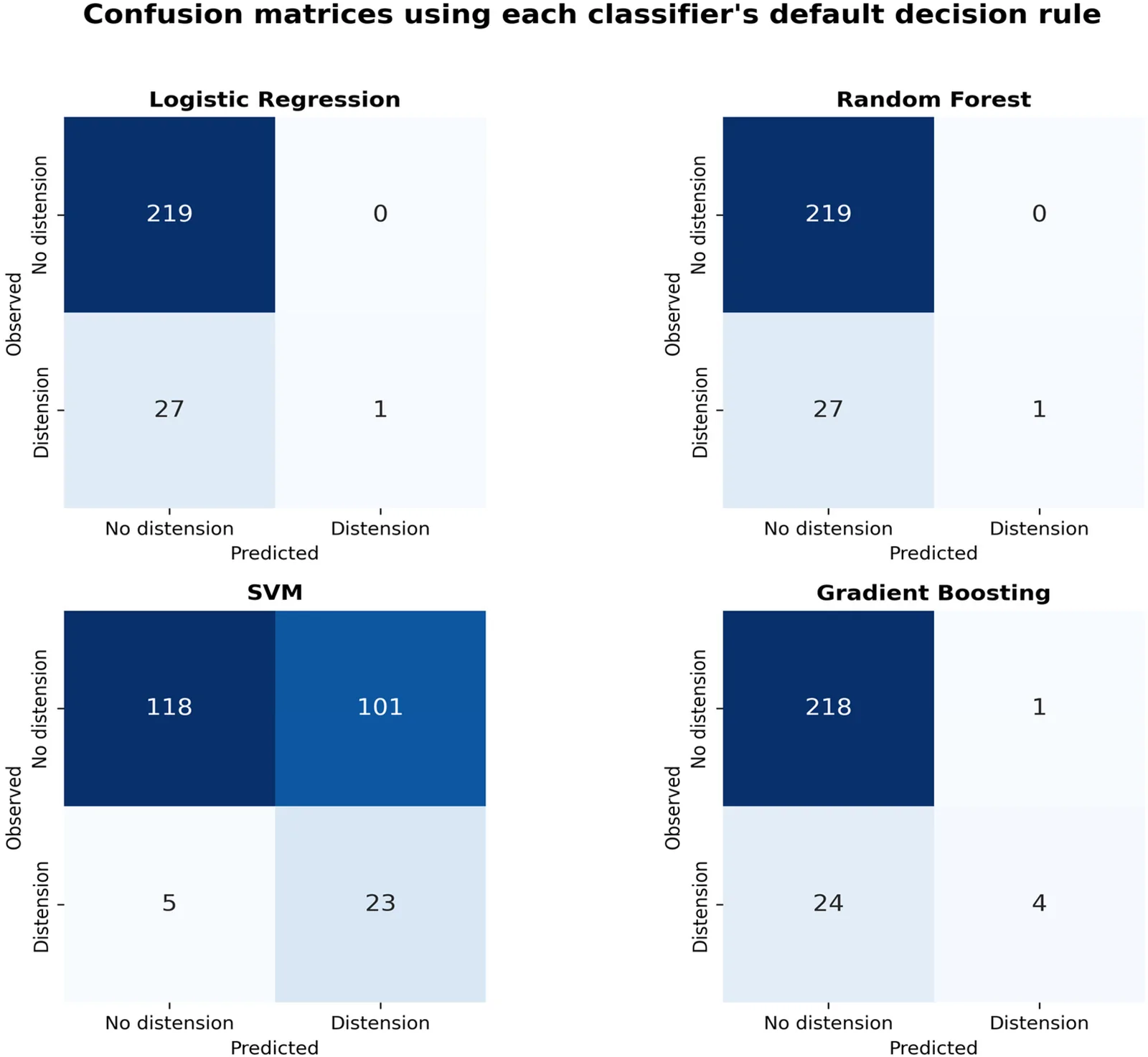
Confusion matrices for the four classifiers using each model's native default decision rule.

**Figure 3:**
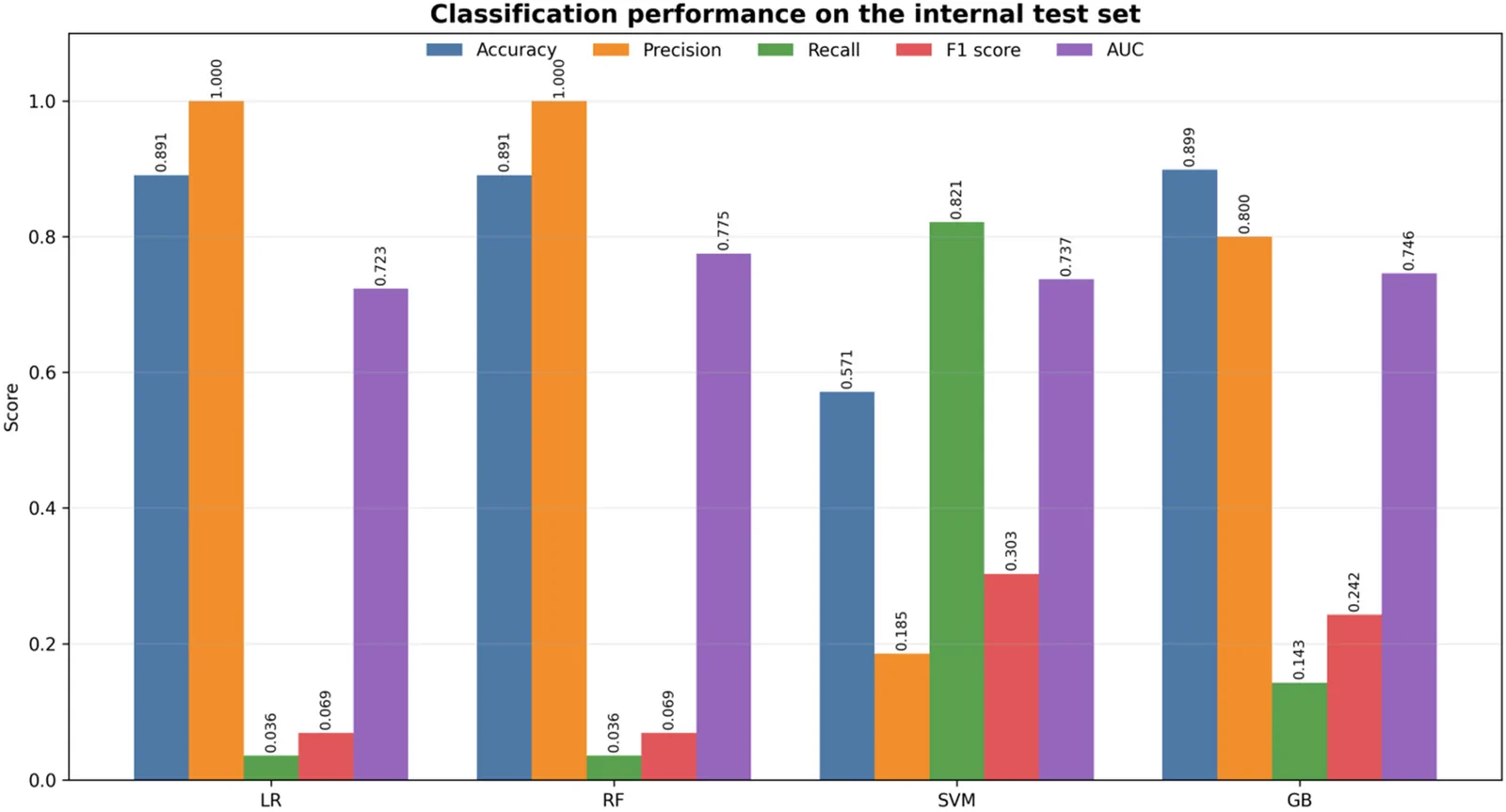
Comparison of accuracy, precision, recall, F1 score, and AUC on the internal test set.

The default classification rules produced different clinical trade-offs (Figures 2, 3). SVM had the highest recall (0.821) but low precision (0.185) and accuracy (0.571). GB had accuracy of 0.899, precision of 0.800, recall of 0.143, and F1 score of 0.242. LR and RF each classified only one event as positive, yielding precision of 1.000 but recall of 0.036; thus their high accuracy (0.891) primarily reflected the class imbalance.

**Figure 4:**
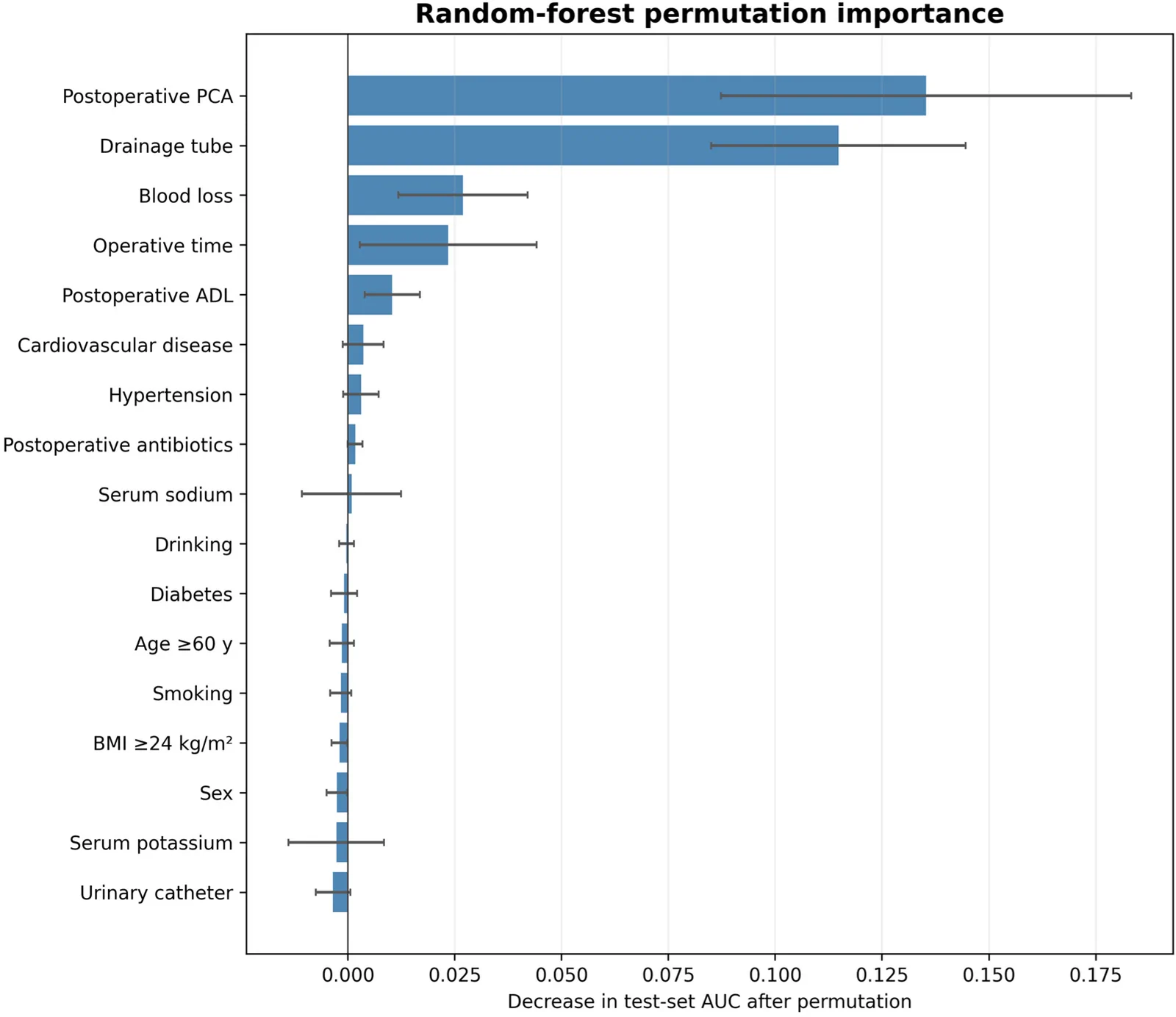
RF permutation importance measured as the mean decrease in internal test-set AUC across 100 repeated permutations. Error bars show the standard deviation across repetitions.

Random-forest permutation importance ranked PCA and surgical drainage tube placement highest, followed by BL, OT, and ADL (Figure 4). Postoperative antibiotic use was retained in all models as requested but produced a comparatively small decrease in AUC when permuted. Negative or near-zero permutation values for several variables indicate no measurable incremental test-set contribution in this fitted model, not evidence that those factors are clinically irrelevant.

### 3.3 TreeSHAP model interpretation

**Figure 5:**
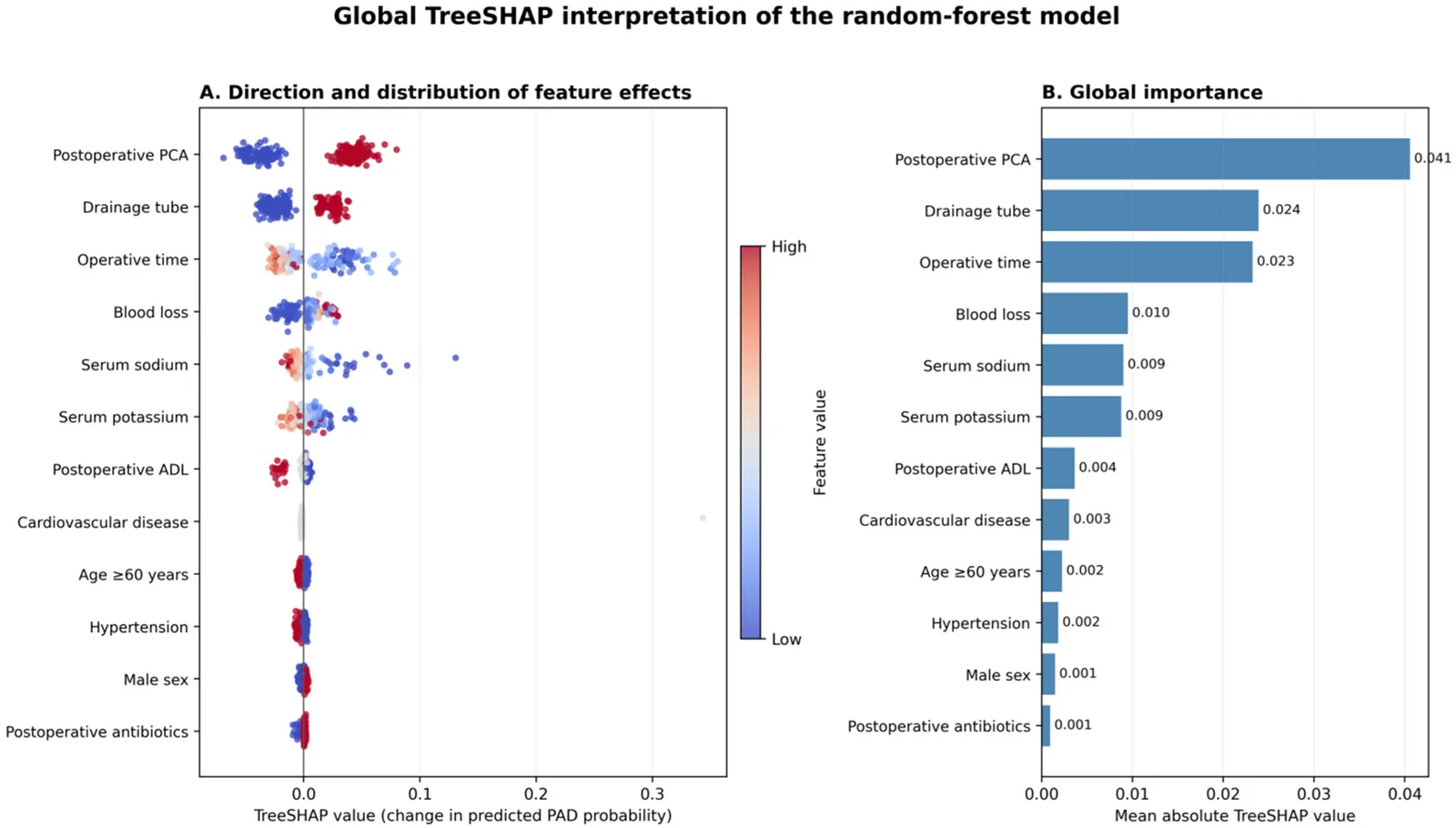
Global TreeSHAP interpretation of the random-forest model in the internal test set (*n* = 247). **(A)** Each point represents one patient; the horizontal position is the feature contribution to predicted postoperative abdominal distension (PAD) probability, and color indicates the observed feature value from low (blue) to high (red). Positive TreeSHAP values increase and negative values decrease the model-predicted PAD probability. **(B)** Global feature importance ranked by mean absolute TreeSHAP value.

**Figure 6:**
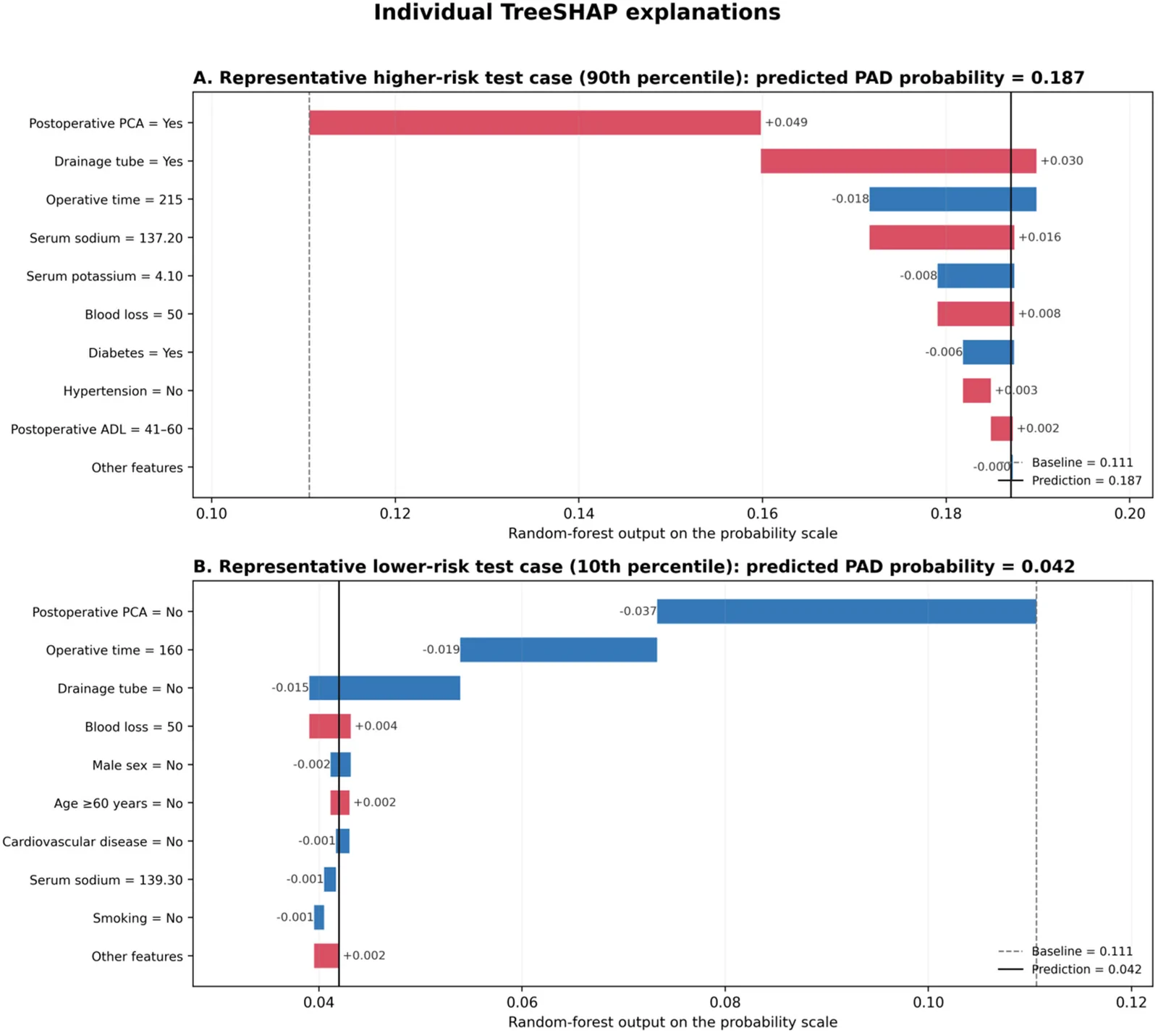
Individual TreeSHAP waterfall explanations for representative internal-test observations nearest the **(A)** 90th and **(B)** 10th percentiles of random-forest predicted risk. Red contributions increase and blue contributions decrease the prediction relative to the model baseline probability of 0.111; the remaining low-magnitude variables are aggregated as “Other features”.

The random-forest expected positive-class probability was 0.111, and TreeSHAP reconstructed every internal-test prediction (maximum absolute additivity error, 2.22 × 10–16). The leading contributors by mean absolute TreeSHAP value were PCA (0.041), surgical drainage tube placement (0.024), OT(0.023), BL(0.010), Na^+^ (0.009), K^+^ (0.009), and postoperative ADL (0.004) (Figure 5). PCA use and drainage placement generally shifted predictions upward; higher OT, electrolyte values, and postoperative ADL generally shifted them downward. Postoperative antibiotic use contributed comparatively little (0.001). The representative observations nearest the 90th and 10th percentiles had predicted PAD probabilities of 0.187 and 0.042, respectively. In the higher-risk example, PCA (+0.049), drainage placement (+0.030), and Na^+^ (+0.016) were partly offset by OT (−0.018) and K^+^ (−0.008). In the lower-risk example, no PCA use (−0.037), OT (−0.019), and no drainage placement (−0.015) dominated (Figure 6). But,these decompositions do not imply that changing a factor would change outcome risk.

## 4 Discussion

### 4.1 Differences in model performance and adaptability to small-sample datasets

In this dataset of 1,234 patients,we dicovered that postoperative abdominal distension occurred in 11.18%. RF had the highest test-set AUC (0.775), indicating moderate discrimination, while the other models had AUCs from 0.723 to 0.746. The DeLong comparison supported a modest AUC difference between RF and GB, but not between the remaining model pairs. Permutation importance and TreeSHAP converged in identifying PCA and drainage tube placement as dominant contributors to the fitted RF model. TreeSHAP added directionality: PCA use and drainage tube placement generally increased the predicted PAD probability, whereas higher postoperative ADL generally decreased it. PCA may act as a marker of postoperative opioid exposure and pain-management intensity, drainage placement may reflect surgical complexity, and lower postoperative ADL may indicate impaired mobility during gastrointestinal recovery. On the other hand，the inverse TreeSHAP patterns observed for OT and electrolyte values were model-specific and may reflect nonlinearities, interactions, confounding, or limited event counts rather than protective biological effects (32–34). Individual waterfall plots can make the model's reasoning more transparent to nursing teams, we suggest that they are pure explanatory outputs rather than treatment recommendations (31, 34).

Previous studies have generally examined postoperative ileus rather than the broader and often earlier clinical phenotype of postoperative abdominal distension. In some systematic reviews and spine-surgery cohorts have associated postoperative ileus with multilevel or complex procedures, longer operative duration, greater blood loss, perioperative opioid exposure, fluid or electrolyte imbalance, and reduced early ambulation (11, 35–37). Our present findings partly align with that literature: PCA use and drainage tube placement may serve as markers of opioid exposure, pain-management intensity, and surgical complexity, while lower postoperative ADL may reflect impaired mobility during gastrointestinal recovery. However, the inverse TreeSHAP patterns observed for operative time and electrolyte values were specific to the fitted model and should not be interpreted as protective biological effects. Differences in outcome definition, assessment timing, case mix, predictor coding, and the limited number of test-set events may account for these patterns (38).

The incremental contribution of this study is therefore methodological and interpretive rather than evidence that machine learning is inherently superior to conventional regression. Earlier spine-surgery studies predominantly used conventional association models, and broader methodological reviews have found that apparent machine-learning advantages may reflect design and reporting differences (26, 27). In contrast,our study compared four algorithms on the same internal test observations and evaluated discrimination, calibration, Brier score, decision-curve net benefit, permutation importance, and patient-level TreeSHAP explanations (39–41). Nevertheless, RF achieved only moderate discrimination, and most pairwise AUC differences were not statistically significant. Therefore，the model should be viewed as an internally evaluated early-postoperative surveillance framework whose incremental clinical value remains to be established through prospective temporal validation, external validation, recalibration, and impact assessment.

A clinically useful prediction model must provide reliable probabilities and an acceptable sensitivity–specificity trade-off, not merely a high AUC or accuracy (42, 43). In our study, default decision rules produced sharply different patterns: SVM emphasized sensitivity at the cost of many false positives, whereas the other models had high specificity but low sensitivity. We therefore considered Calibration, Brier score, and decision curve analysis alongside discrimination. Before implementation, a clinically justified threshold should be selected and validated prospectively according to the intended nursing action and the relative consequences of missed cases and false alerts.

### 4.2 Temporal scope and potential postoperative data leakage

Postoperative ADL, PCA, and postoperative antibiotic use were deliberately retained as early postoperative predictors. We coded postoperative antibiotic exposure only as use (yes/no), and duration was excluded. Our dataset contained a separate field for the onset day of postoperative abdominal distension for all 138 event cases. Most events occurred after the day of surgery (126/138, 91.3%): 64 occurred on D1, 32 on D2, 8 on D3, and 22 from D4 to D18; 12 cases (8.7%) were recorded on D0. This onset-day variable provides a temporal anchor and supports interpreting postoperative ADL, PCA use, and antibiotic use within an early postoperative surveillance window rather than as predictors in a purely preoperative risk model.

However, the analysis dataset records postoperative day rather than exact clock times for predictor assessment or treatment initiation and symptom onset. The recorded onset day reduces, but does not eliminate, the possibility of postoperative data leakage, reverse temporal ordering, or treatment-response bias. Accordingly, we interpreted models should as exploratory dynamic surveillance models.

### 4.3 Strengths, limitations, and future directions

This study addressed a clinically relevant postoperative nursing problem, we analyzed a sum of dataset of 1,234 patients, retained all 17 prespecified variables, restricted preprocessing and hyperparameter selection to the training workflow, formally compared correlated AUCs, and evaluated calibration, Brier score, decision curves, permutation importance, and exact TreeSHAP explanations. By reporting the complete grids, selected hyperparameters, software versions, corrected test-set results, and verified additive explanations improves reproducibility and transparency (21, 44–46).

We acknowledged that there are some limitations. First, the retrospective single-center design may introduce selection bias, information bias, and residual confounding. Second, external validation was unavailable, and the internal test set contained only 28 events, producing wide confidence intervals and unstable classification metrics. Third, postoperative abdominal distension was identified from routine clinical and nursing documentation rather than a prospectively standardized tool. Fourth, although the postoperative day of abdominal-distension onset was available, exact within-day timestamps for postoperative ADL assessment, PCA initiation, antibiotic initiation, and symptom onset were unavailable, leaving residual potential for temporal leakage or reverse causation. Fifth, the model used a single split and native default classification rules; threshold optimization, repeated internal validation, recalibration, and clinical impact evaluation remain necessary. Finally, permutation importance and TreeSHAP describe the behavior of this fitted model in the internal test cohort; but both can not establishe causal importance, thereofor we suggest that individual explanations should not guide care before prospective and external validation.

Future research should prospectively standardize and timestamp abdominal-distension assessment and all postoperative predictors, externally validate and recalibrate the models in independent multicenter cohorts, compare pre/intraoperative-only models with early postoperative models, and select action thresholds according to nursing workflow and the clinical costs of false negatives and false positives (47, 48).

## 5 Conclusion

Our analysis demonstrated that RF showed the highest internal AUC (0.775), but discrimination was moderate and most pairwise AUC differences were not statistically significant. Permutation importance and TreeSHAP consistently identified PCA and drainage tube placement as leading random-forest contributors, and TreeSHAP provided directionally explicit global and patient-level explanations. Eventhough，postoperative antibiotic use was included as a binary predictor and had smaller incremental importance. Because these explanations are predictive rather than causal and performance varied markedly across native decision rules, no model is ready for routine bedside use. Prospective temporal validation, external validation, recalibration, and clinically justified threshold selection are required as future study.

## Data Availability

The raw data supporting the conclusions of this article will be made available by the authors, without undue reservation.
