# Supplementary material for: Machine learning-based risk prediction model for postoperative abdominal distension after spinal surgery under general anesthesia
Source: Front Med (Lausanne). 2026 Sep 4;13:1871136. doi: 10.3389/fmed.2026.1871136 (PMC13585623; doi:10.3389/fmed.2026.1871136)
Supplement: Supplementary file 1 [file Supplementaryfile1.pdf]

Supplementary Material

Machine learning-based risk prediction model for postoperative abdominal distension after spinal surgery under general anesthesia

Supplementary Table S1

Hyperparameter search grid used for model tuning.

Model	Hyperparameters evaluated	Selected values
LR	penalty: l1, l2; C: 0.01, 0.1, 1, 10, 100; solver: liblinear; class_weight: None, balanced	C=1; l1; class_weight=None
RF	n_estimators: 200, 500; max_depth: None, 5, 10, 20; min_samples_split: 2, 5, 10; min_samples_leaf: 1, 2, 4; max_features: sqrt, log2; class_weight: None, balanced	200 trees; depth=5; split=5; leaf=1; sqrt; no class weight
SVM	kernel: linear, rbf; C: 0.1, 1, 10, 100; gamma: scale, auto; class_weight: None, balanced; probability=True	linear; C=0.1; gamma=scale; balanced
GB	n_estimators: 100, 200, 300; learning_rate: 0.01, 0.05, 0.1; max_depth: 2, 3, 4; min_samples_split: 2, 5, 10; subsample: 0.8, 1.0	300 trees; rate=0.01; depth=4; split=5; subsample=0.8

Supplementary Table S2

Software and package versions used for model development and statistical analysis.

Software/package	Version
Python	3.12.13
numpy	2.3.5
pandas	2.2.3
scikit-learn	1.8.0
scipy	1.17.0
matplotlib	3.10.8
seaborn	0.13.2
